# Self-calibrated acceleration and detail preserving for semantic segmentation of lactating sows and piglets under low-light conditions

**DOI:** 10.1038/s41598-025-09146-0

**Published:** 2025-07-03

**Authors:** Aqing Yang, Yueju Xue, Na Han, Jiabi Zheng, Lei Zhang, Yizhi Luo

**Affiliations:** 1 College of Computer Science, Guangdong Polytechnic Normal University, Guangzhou, 510665 China; 2 College of Electronic Engineering, South China Agricultural University, Guangzhou, 510642 China; 3 Institute of Facility Agriculture, Guangdong Academy of Agricultural Sciences, Guangzhou, 510640 China; 4 State Key Lab. for Novel Software Technology, Nanjing University, Nanjing, 210008 China

**Keywords:** Low-light image enhancement, Semantic segmentation, Lactating sows and piglets, Smart animal husbandry, Self-calibrated acceleration, Semantic perceptual loss, Image processing, Machine learning

## Abstract

Pixel-wise semantic segmentation of lactating sows and piglets is critical to explore maternal traits in smart livestock breeding and production. However, semantic segmentation algorithms do not yield satisfactory results under low-light conditions because these methods are highly dependent on image quality. Therefore, an efficient and detail-preserving low-light image enhancement is necessary and crucial for animal monitoring under low-light conditions. In this paper, a low-light enhancement method called self-calibrated acceleration and detail preserving (SADP) was proposed to improve the semantic segmentation performance of lactating sows and piglets. Specifically, a self-calibration acceleration module that accelerated the convergence among all stages was proposed to improve the computational efficiency and a semantic perceptual loss term was proposed for a high detail and semantic information preservation. Plenty of experiments demonstrated that SADP outperformed the existing well-known methods in both visual quality and efficiency (-0.013s in execution time from 0.0163s to 0.0033s, -0.0031 M in mode size from 0.0034 M to 0.0003 M), and even more improved the performance of semantic segmentation of lactating sows and piglets, raising the mean IOU from 0.8686 to 0.8872. Obviously, SADP also can be used to improve the performance of other high-level visual tasks. This may build a good foundation for the following visual tasks and further promote the livestock breeding and production.

**Supplementary Information:**

The online version contains supplementary material available at 10.1038/s41598-025-09146-0.

## Introduction

Under smart animal husbandry, machine vision tasks such as object detection^1,2^ image segmentation^3,4^ behavior recognition^5,6^ etc., have been widely used, which facilitate livestock farmer in automatic surveillance, in further to promote the livestock breeding and production. One of keys to obtain satisfactory results is that these visual algorithms are built on a set of high quality images. Especially for pixel-wise segmentation task, the segmentation result is highly dependent on the image/video quality. Unfortunately, in actual livestock environment, especially non-standard, small and medium-sized livestock farms, images or videos captured in low light conditions usually suffer from severe information loss and low contrast. These low-light images or videos greatly degrade the performance of high-level visual tasks especially pixel-wised semantic segmentation task, and bring great difficulty in video surveillance^7–9^ though human eye observation. Therefore, low-light enhancement is inevitable before further data interpretation. This paper takes low-light images captured from pig farm as our research object, aiming at proposing a novel effective image enhancement method that can reveal the information hidden in the dark, which can benefit the performance of semantic segmentation even more other high-level visual tasks. Next, we analyze and summarize the development process of low-light image enhancement, and further proposed the main work of this paper.

Low-light image enhancement techniques (LIE) mainly consist of traditional and deep learning approaches. Traditional LIE methods include histogram equalization (HE), Gamma correction^10–12^ Retinex model^13–16^ etc. Among them, the Retinex theory-based methods are the most prevalent and many scholars pay attention to its improvement. Retinex theory-based methods decompose a general image into two components with priors and regularization, where one component reflects the illumination and the other refers to the reflection. Generally, the reflection component is treated as the enhanced results which reduce or even remove the influence of illumination and preserve the reflection properties of the object itself. However, the reflection component suffers from detail loss, color distortion, noise and other problems due to that the reflection component is obtained by approximate priors estimations.

Currently, deep learning has been widely used in various fields of visual tasks such as animal identification^17^ and behavior recognition^18^ from pictures or videos, and it achieved unprecedented success^19–21^. Unsurprisingly, CNN-based models for LIE problem have made great achievements. As for LIE task, most of CNN-based models^22–25^ are based on supervised learning, which relied on paired data for supervised training. Actually, it is difficult to collect the paired images in smart animal husbandry, limited by different lighting conditions of the unstructured environment. Also the visual standard of the normal light images for a LIE is difficult to define. To solve these issues, unsupervised-based methods have received widely attention recently^26–29^. These methods do not rely on any paired normal light images, so it is more feasible in practical application. Currently, most of unsupervised CNN-based methods tend to enhance the low-light image by designing different network architectures. For example, EnlinghtenGAN^28^ is the first image reconstruction-based methods, which based on Generative Adversarial Network (GAN) structure and can be trained under the unpaired supervision. It introduces an attention module on U-Net to transform the low-light images into a normal condition. Zero-DCE^27^ considers LIE as a problem of image-detailed curve estimation. Thereby, it designed a deep learning-based curve estimation network to produce high-order curves as its enhanced results. SSIENet^26^ established a decomposition approach for LIE based on Retinex theory. RUAS^29^ established a Retinex-based unrolling framework and architecture optimization to construct enhanced image. These well-known unsupervised CNN-based methods trained their network without paired data. However, these methods still have some limitations: (1) Most of the papers did not consider the efficiency of the low-light enhancement methods, while this metric is crucial in the smart animal husbandry applications; (2) Almost all methods only focus on improving visual quality at the expense of image details, which may degrade the performance of downstream tasks.

In the perspective of intelligent animal husbandry, the purpose for improving the image quality is to improve the performance of down stream tasks such as semantic segmentation and behavior recognition in low-light environment. Therefore, this paper developed a novel Retinex-inspired model for fast and high-quality image enhancement that can improve the performance on the semantic segmentation of lactating sows and piglets under low-light conditions. Specifically, first, we proposed a self-calibration acceleration module for fast inference and a novel unsupervised loss for keeping image details that can improve the performance of downstream tasks. Second, we took the proposed method as a pre-processing for semantic segmentation of lactating sows and piglets. Finally, we conducted plenty of experiments including ablation studies and comparative experiments with other methods, to evaluate the performance of our proposed method in image visual quality, computing cost and semantic segmentation.

## Materials

### Ethics declarations

Since the research work of this paper only covered the observational field studies, which did not involve any type of interaction with animals or manipulation of the environment. Therefore, this study did not require ethical approval according to rules of Institutional Animal Care and Use Committee. Confirmation that these pigs were not handled by the authors during the study. Confirmation that all experimental protocols have been approved by Lejiazhuang farm in Foshan City, Guangdong Province, China, where the video data were collected. Confirm that all methods were performed in accordance with relevant guidelines and regulations.

To optimize livestock breeding, this work focused on the research of maternal quality of sows. So we takes the nursing sow and their piglets as our research objects. Data collection was carried out in a real commercial pig farm, located in Foshan city, China. For the convenience of subsequent work on maternal behavior recognition, farrowing pens were selected as our experimental conditions, where each pen was housed one mother sow and 8–10 piglets.

**Fig. 1:**
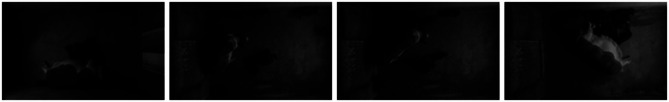
The captured low-light images under different illuminations.

An RGB camera (HIKVISION DS-2CD1321D-I) was placed above the farrowing pen to capture images. The captured images under low-light conditions shows in Fig. 1. Due to minimal brightness variations under low-light conditions, we only selected 2772 low-light images with different brightness levels that were captured at different time periods. Also, these images were captured from different farrowing pens to demonstrate the generalization capability of the algorithms. The size of raw images was 1920 × 1080 and then resized to 960 × 540 to avoid GPU exhaustion.

All the images were divided into two parts. One part with 1865 images was selected for SADP training and testing, and the other part with 907 images was used for semantic segmentation to further verify the performance of SADP for downstream tasks. The details for the experimental data were described in Table 1, where 1465 samples were used for training, and the remaining were used for testing. In addition, another 607 and 300 images were selected for training and testing of the down-streamed semantic segmentation tasks.

**Table 1:** Details of dataset split for our experiment.

Dataset	Dataset split	Number of images
SADP-D1	Training set for SADP	1465
SADP-D2	Validation set for SADP	400
SEG-D1	Training set for Semantic Segmentation	607
SEG-D2	Validation set for Semantic Segmentation	300

## Methods

**Fig. 2:**
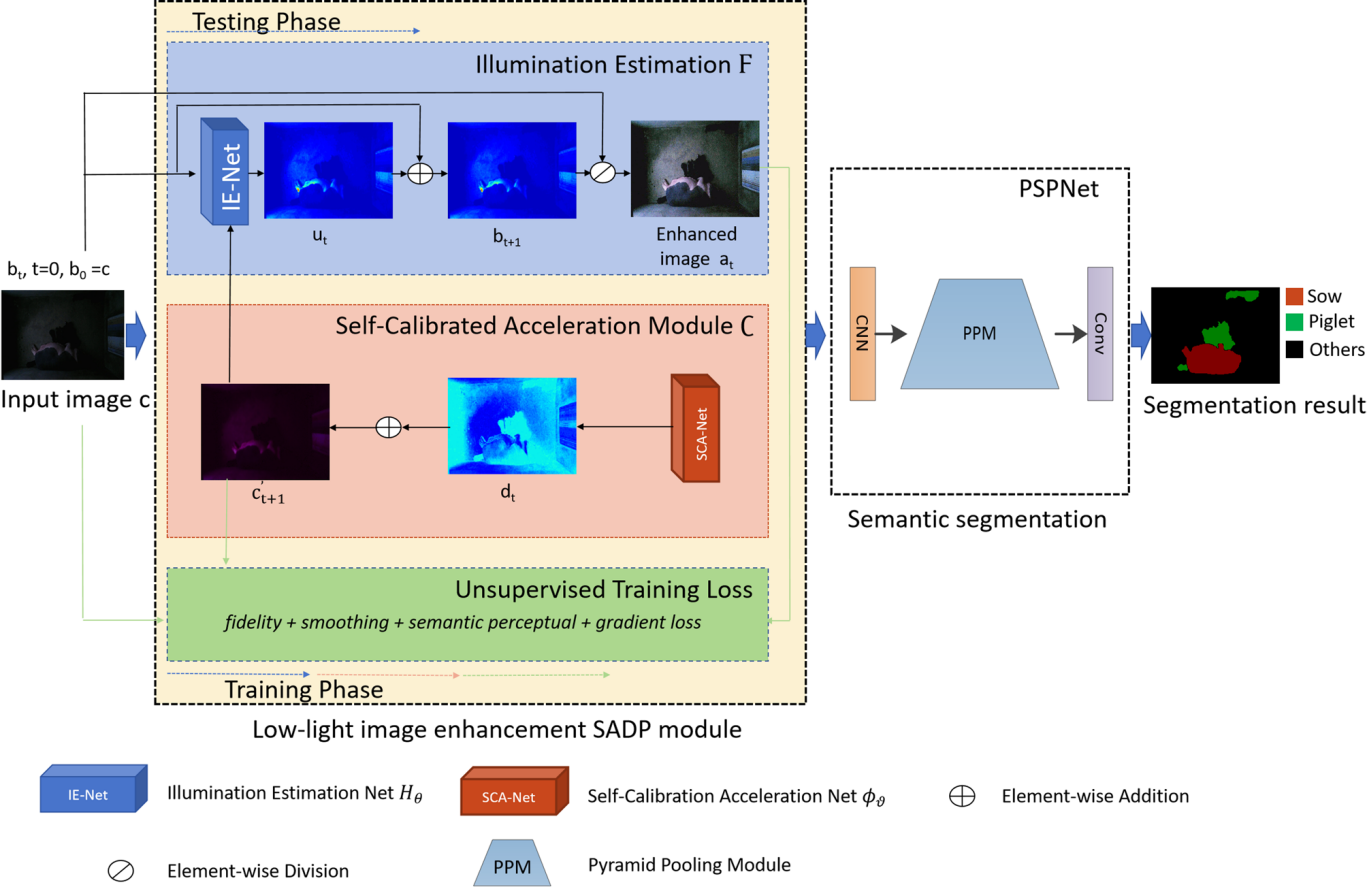
The overall structure of proposed SADP.

Figure 2 presents the overall flowchart of this work. It consists of two main components: low-light image enhancement and semantic segmentation. To address the issues of computational burden and detail loss, a self-calibrated acceleration and detail preserving enhancement (SADP) was proposed by designing a self-calibrated acceleration module and unsupervised loss to enhance image under low light conditions. After that, the proposed image enhancement method SADP was taken as a pre-processing for further semantic segmentation of lactating sows and piglets.

### Low-light image enhancement

Based on Retinex theory^30^ the proposed SADP module designed a self-calibrated acceleration module and a semantic perceptual loss term to address the issues of computational burden and detail loss. The overall structure of the proposed SADP was shown in Fig. 2, where, the upper is a classical multi-stage optimization process of Retinex model, the middle is the proposed self-calibrated acceleration module and the bottom is the unsupervised training loss.

In this section, we firstly introduced the backbone architecture of the developed multi-stage optimization process of Retinex mode and illumination learning with weight sharing. Next, we proposed a self-calibrated acceleration module to address the problem of computational burden caused by the multi-stage learning pattern. Also, we introduced a novel perception loss design to preserve more image details which may improve the performance of the downstream visual tasks. Finally, we took the proposed image enhancement method as a pre-processing for further semantic segmentation of lactating sows and piglets.

#### Retinex-inspired illumination Estimation with weight sharing

**Fig. 3:**
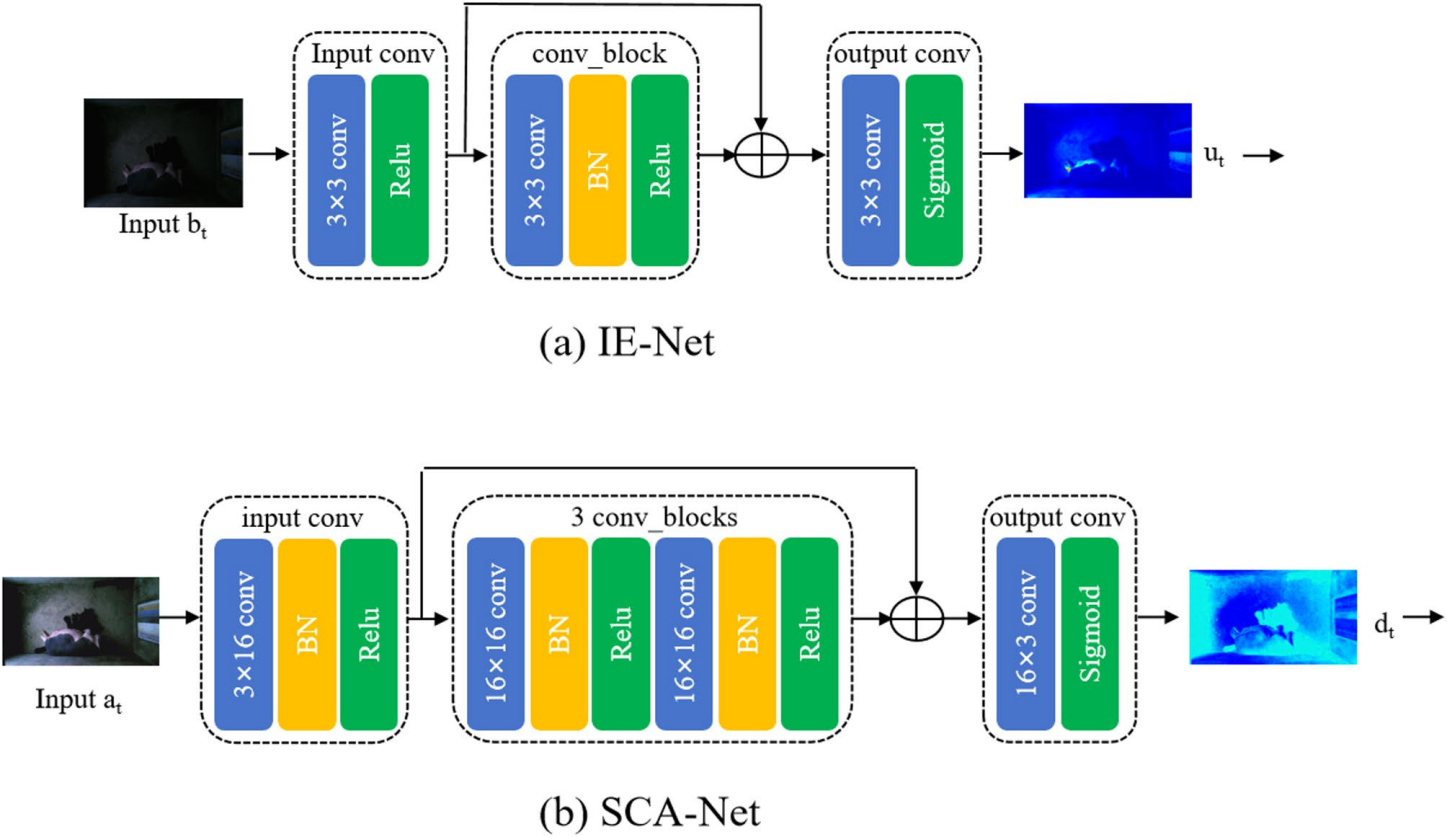
The architecture of Illumination Estimation Net(IE-Net) and Self-Calibration Acceleration Net(SCA-Net).

Our method was built upon Retinex theory which divides an image into reflection and illumination components:

1 $$\:{c}\:=\:{a}\:\otimes\:{b}$$

where c is the captured original low-light image. b is illumination image and a is the reflection image that usually is treated as a clear enhanced image. The operator $\otimes$ represents the element-wise multiplication. In general, the illumination image and the original image are similar on content structure, semantic distribution or with linear correlation. Therefore, illumination image can be estimated from the original image. Based on this theory, our method aims to learn the illumination image b indirectly from the captured low-light image c, further obtaining the desired clear image a by removing the illumination image. Inspired by multi-step optimization for LIE^29,31^ here a residual mapping $\:{{\rm\:H}}_{\theta\:}$ with learnable parameters $\:{\uptheta\:}$ was introduced to learn a residual representation between the illumination and low-light image. The residual representation offered a progressive strategy to the muti-stage LIE task. The process was shown in blue box in Fig. 2, it can be modeled as:

2 $$\:\text{F}\left({\text{b}}_{\text{t}}\right):\left\{\begin{array}{c}{\text{u}}_{\text{t}}\:=\:{{\rm\:H}}_{\theta\:}\left({\text{b}}_{\text{t}}\right),\:{\text{b}}_{0}=\:c,\\\:{\text{b}}_{\text{t}+1}\:=\:{\text{b}}_{\text{t}}+{\text{u}}_{\text{t}},\end{array}\:\right.$$

where $\:{\text{u}}_{\text{t}}$ and $\:{\text{b}}_{\text{t}}$ denote the residual and illumination component at the t-th (t = 0,…,T-1) stage, respectively. The residual mapping $\:{{\rm\:H}}_{\theta\:}$ was modeled by a CNN network abbreviated as IE-Net as shown in Fig. 2. Note that during multi-stage illumination optimization process, we adopt a same architecture $\tt H$ and weight $\:{\uptheta\:}$ at each stage. IE-Net only consisted of 3 × 3 convolution + Relu, 3 × 3 convolution + BN + Relu and layers, 3 × 3 convolution + Sigmoid layers as shown in Fig. 3(a), which guaranteed the scale in-variance of image. In addition, the simple convolution operation with activation function and feature fusion mechanism preserved the image content and structure as much as possible. The hyper parameters setting for the IE-Net in this paper were given in the upper part of Table 2. Compared with directly illumination estimation from a low-light image, learning a residual representation guarantees illumination performance and improve the steadiness.

During multi-stage illumination estimation process, a low-light image b_0_ (b_0_ = c) was as the input of the first stage and was sent to illumination estimation model, and outputting an illumination map b_1_ as the input of the next stage for a further illumination estimation. According to this iterative mechanism, the refined illumination image was obtained, then using the formula below, a clear image can be obtained:

3 $$\:{\text{a}}_{\text{t}}\:=\:\text{c}\oslash\:{\text{b}}_{\text{t}}\:$$

where $\:\oslash\:$ represents the element-wise division. According the the defining of the illumination estimation module, $\:{\text{a}}_{\text{t}}$ is the clear image at t-th stage, where the output $\:{\text{a}}_{\text{t}}$ of the last stage is considered as the desired clear image with the best quality.

**Table 2:** The hyper parameter setting for the network architecture in this paper.

Layer	Input/output	Output size	Input/C	Output/C
**Details of IE-Net**				
input conv	$\:{\text{b}}_{\text{t}}$/IE_f1	960 × 540	3	3
conv_blocks	IE_f1/IE_f2	960 × 540	3	3
fusion	IE_f1, IE_f2/IE_f3	960 × 540	3	3
output conv	IE_f3/$\:{\text{u}}_{\text{t}}$	960 × 540	3	3
**Details of SCA-Net**				
input conv	$\:{\text{a}}_{\text{t}}$/SCA_f1	960 × 540	3	16
3 conv_blocks	SCA_f1/SCA_f2	960 × 540	16	16
fusion	SCA_f1, SCA_f2/SCA_f3	960 × 540	16	16
output conv	SCA_f3/d_t_	960 × 540	16	3

Note that the cascade architecture inevitably brings inference error due to the weight sharing between different blocks. To cope this problem, a self-calibrated acceleration module was proposed to accelerate convergence speed of the first stage and achieved the same quality level of output for each stage. In this way, only the first stage need be used for illumination estimation that can largely accelerate the inference speed.

#### Self-calibrated acceleration module

To solve the problem of inference speed cost mentioned above, here, we proposed a self-calibrated acceleration module which aims to make the output of each stage convergent to the same state. In the illumination estimation module, the input of each stage (except for the first stage) stems from the output of the previous stage, where the low-light image served as the input of the first stage. Based on this pattern, we can connect each stage in the network to the input image which may simply the inference framework. It can be presented as:

4 $$\:\mathcal{C}\left({\text{b}}_{\text{t}}\right):\left\{\begin{array}{c}{\text{a}}_{\text{t}}\:=\:c\oslash\:{\text{b}}_{\text{t}}\\\:{\text{d}}_{\text{t}}\:=\:{\varphi\:}_{\vartheta\:}\left({\text{a}}_{\text{t}}\right)\\\:{\text{c}}_{\text{t}}^{{\prime\:}}\:=\:c+{\text{d}}_{\text{t}}\end{array}\right.$$

where $\:{\text{d}}_{\text{t}}$(t > 0) represents a self-calibrated map that was added to the low-light image c to present the difference between the low-light and the input in each stage. After that, adding $\:{\text{d}}_{\text{t}}$, $\:{\text{c}}_{\text{t}}^{{\prime\:}}$ as the converted input for each stage will correct the input of each stage to the same state. $\:{\varphi\:}_{\vartheta\:}$ is the mapping function in which $\:{\upvartheta\:}$ is a learnable parameter. It was modeled by a CNN network abbreviated as SCA-Net as shown in Fig. 1. After using the calibrated module, the operation in each stage (except the first stage, t > 0) can be defined as:

5 $$\:\text{F}\left({\text{b}}_{\text{t}}\right)\to\:\text{F}\left(\mathcal{C}\right({\text{b}}_{\text{t}}\left)\right)$$

Like the IE-Net, SCA-Net adopted a same architecture and weight sharing mechanism during multi-stage training. The architecture of SCA-Net was shown in Fig. 3(b). Compared with the IE-Net, SCA-Net increased channels and network depth, which aims at extracting more subtle differences between the low-light image and the input in each stage. The details about the input/output in each layer of the SCA-Net were described in the lower part of Table 2.

#### Unsupervised training loss

As we know, it is difficult to obtain the reference image for each low-light image in the smart animal husbandry applications. Here, an unsupervised loss function was designed to train the network. Additionally, we proposed semantic perceptual and gradient loss terms to save more details and reduce noise. Finally, the proposed loss function consisted of four loss terms, which can be expressed as:

6 $$\:L\:=\:\alpha\:{L}_{f}+\beta\:{L}_{s}+{L}_{sem}+{L}_{\nabla\:}$$

where $\:{L}_{f}$,$\:{\:L}_{s}$, $\:{L}_{sem},\:{L}_{\nabla\:}$ represent the fidelity, smoothing, semantic perceptual and gradient loss, respectively. $\:\alpha\:$ and $\:\beta\:$ are weighting coefficients. The specific description of each Loss term is as follows.

$\:{L}_{f}$ is a fidelity item that aims to minimize the difference between the illumination component and the input of each stage. It is presented as:

7 $$\:{L}_{f}\:=\:\sum\:_{\text{t}=1}^{\text{T}}{\parallel{\text{b}}_{\text{t}}-(\text{c}+{\text{d}}_{\text{t}-1})\parallel}^{2}$$

where T is the total stage number. According to the definition above, $\:\text{c}+{\text{d}}_{\text{t}-1}$presents the corrected input and $\:{\text{b}}_{\text{t}}$ presents the estimated illumination in t-th stage.

$\:{L}_{s}$ is the illumination smooth loss which is a broad consensus in LIE task^27,32,33^. It guarantees that the lighting property of the illumination map is smooth in space, which can be presented as:

8 $$\:{L}_{s}=\sum\:_{i=1}^{N}\sum\:_{j\in\:N\left(i\right)}{w}_{i,j}\left|{\text{b}}_{\text{t}}^{i}-{\text{b}}_{\text{t}}^{j}\right|$$

where *N* is the pixel quantity. and $\:i$ is the pixel index. $\:N\left(i\right)$ presents the pixels in a 5 × 5 window centered on pixel $\:i$. $\:{w}_{i,j}$ refers to the weight calculated by $\:{w}_{i,j}=exp(-\frac{{\sum\:}_{ch}{\left(\right({\text{c}}^{i,ch}+{\text{c}}_{\text{t}-1}^{i,ch})-({\text{c}}_{i,ch}+{\text{c}}_{\text{t}-1}^{j,ch}\left)\right)}^{2}}{2{\sigma\:}^{2}})$, where *ch* is the index to the channel in the YUV space.

$\:{L}_{sem}$ is the semantic perceptual loss to maximize the similarity between the illumination component and the input of each stage in feature level. It is calculated in feature space of VGG^34^ presented as:

9 $$\:{L}_{sem}\:=\:\sum\:_{\text{t}=1}^{T}{\parallel{\mathcal{V}(\text{b}}_{\text{t}})-\mathcal{V}(\text{c}+{\text{d}}_{\text{t}-1})\parallel}^{2}$$

where $\:\mathcal{V}$ presents VGG network, $\:{\mathcal{V}(\text{b}}_{\text{t}}\left)\:\text{a}\text{n}\text{d}\:\mathcal{V}\right(\text{c}+{\text{d}}_{\text{t}-1})$ is the deep representation extracted from $\:{\text{b}}_{\text{t}}$ and $\:\text{c}+{\text{d}}_{\text{t}-1}$, respectively. The semantic perceptual loss further ensures the fidelity in the image details.

Low-light image enhancement generally introduces a lot of noise. To suppress noise, image gradient loss $\:{L}_{\nabla\:}$ was introduced. Based on the correlation of the noises and the gradients, we minimized the $\:{L}_{\nabla\:}$ to suppress noise, which can be presented as:

10 $$\:{L}_{\nabla\:}=\sum\:_{\text{t}=1}^{T}{\parallel\nabla\:{a}_{\text{t}}^{x}\:+\:\nabla\:{a}_{\text{t}}^{y}\parallel}^{1}$$

where $\:\nabla\:{a}_{\text{t}}^{x}$ and $\:\nabla\:{a}_{\text{t}}^{y}$ represent the gradient in *x* and *y* directions of the image produced in t-th stage.

The final unsupervised loss function $\:{L}_{f}\:$for training the whole of network utilizes the corrected input $\:\text{c}+{\text{d}}_{\text{t}-1}$ to constrain illumination output, which solves the difficulty of obtaining ground truth, and $\:{L}_{f}$ guarantees the image quality from content, semantic perceptual, illumination smooth and noise.

### Semantic segmentation for lactating sows and piglets

#### Semantic segmentation method

Semantic segmentation can obtain pixel level semantic information, nowadays it is widely applied to the application of smart livestock-farming, such as body size estimation and behavior recognition. However, under low-light conditions, it is difficult to obtain a satisfactory result in semantic segmentation. In this paper, SADP as an image pre-processing method, and the enhanced images were fed into a segmentation network to improve the segmentation performance. Here, considering the capability of segmentation network PSPNet^35^ in integrating local and global context efficiently, we selected PSPNet to segment sows and piglets from low-light images. In addition, to balance the complexity of the model and computational efficiency in our task, as well as the compatibility of the network with PSPNet, we chose resnet50 as the backbone. To verify the effectiveness of SADP, we did not make any changes to the structure and parameters of PSPNet.

#### Evaluation metrics for semantic segmentation

The mean Intersection over Union (mIOU) was employed to evaluate the effectiveness of the semantic segmentation method. mIOU measures the average overlap ratio between predicted segmentation masks and ground truth across all classes, which provides a comprehensive way to evaluate segmentation performance while accounting for all classes equally. Therefore, mIOU is the most widely used evaluation metric for semantic segmentation tasks. The metric first calculates the Intersection over Union (IOU) for each individual class, then computes the mean value across all classes. The IOU for class *C* and mIOU are formulated as :

11 $$\:{IOU}_{C}=\frac{{TP}_{C}}{{TP}_{C}+{FP}_{C}+{FN}_{C}}$$

12 $$\:mIOU=\frac{1}{C}\sum\:_{C=1}^{C}{IOU}_{C}$$

Where $\:{TP}_{C}$ represents true positives for class *C.* $\:{FP}_{C}$ represents false positives for class *C.* $\:{FN}_{C}$ represents false negatives for class *C.*

###  Experimental setup

As described in the Sect. 2, SADP-D1 and SADP-D2, without pairs, were used to train and evaluate the unsupervised SADP model, respectively. During the training process, only the low-light images were employed and the batch size was set to 2. T was set to 3, which had already been proven to achieve promising results. Our module was trained using Adam optimizer with parameters $\:{\beta\:}_{1}=0.9,\:{{\upbeta\:}}_{2}=0.999$, and $\:{\epsilon}={10}^{-8}$. The learning rate was set to $\:{10}^{-4}$ and the epoch number was set to 100. The weighting coefficients $\:\alpha\:$ and $\:\beta\:$ in Eq. (6) were set to 1 and 1.5, respectively by tuning their values to the best. As described in Sect. 3.1.1 and Sect. 3.1.2, IE-Net and SCA-Net adopted 3 and 4 convolution layers to reduce the training workload. All the training of SADP can be finished in 11 h. During the testing process, only IE-Net was used to estimate illumination map. Then, the illumination map at the first stage was used to obtain the desired clear image by dividing illumination from low-light image, which greatly improved the inference speed. It should be noted that the testing data SADP-D2 had not been trained, which may ensure that the experimental results can reflect the generalization capability of the algorithms.

In the segmentation stage, the PSPNet was trained using Adam optimizer with momentum$\:\:=0.9$ and weight-decay = $\:{10}^{-4}$. The learning rate was set to $\:{10}^{-4}$, the epoch number was set to 50 and batch size was set to 4.

The proposed method was evaluated visually and quantitatively and compared with the mainstream LIE approaches. Specifically, five no-reference metrics including HIGRADE-1^36^, HIGRADE-2^36^, BRISQUE^37^ CEIQ^37^ and ENIQA^37^ were used as our evaluated metrics. For HIGRADE-1, HIGRADE-2 and CEIQ, a higher value indicates a better quality, while for BRISQUE and ENIQ, the lower is better. In addition, we applied the enhancement results on the SEG-D1 and SEG-D2 datasets to semantic segmentation for further evaluating the visual performance of our proposed algorithm. All experiments were run using an Nvidia TITAN V GPU with PyTorch framework^38^.

## Results and discussions

**Fig. 4:**
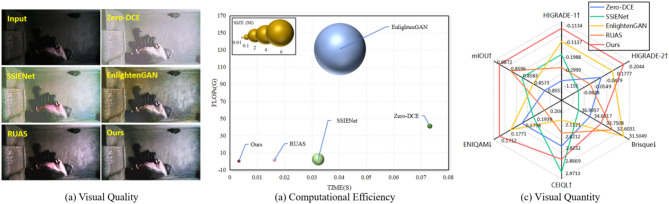
Comparison among recent unsupervised state-of-the-art methods and our method. (**a**) Comparison in enhanced visual quality. (**b**) Comparison in computational efficiency. (**c**) Comparison in non reference image quality evaluation metrics.

This section mainly introduced the experiment results. First, an ablation study was carefully conducted to demonstrate the effectiveness of the proposed modules. Next, we compared the proposed method with the state-of-the-art low light enhancement algorithms, and the comparison covered both the image quality and the influences to the down-streamed semantic segmentation task. The overall experimental results including visual quality, visual quantity and computational efficiency were shown in Fig. 4, where the visual quantity is visualized by a sketch map to clearly describe the comparison results. The specific experimental results are as follows.

### Ablation study

#### Self-calibrated acceleration

**Fig. 5:**
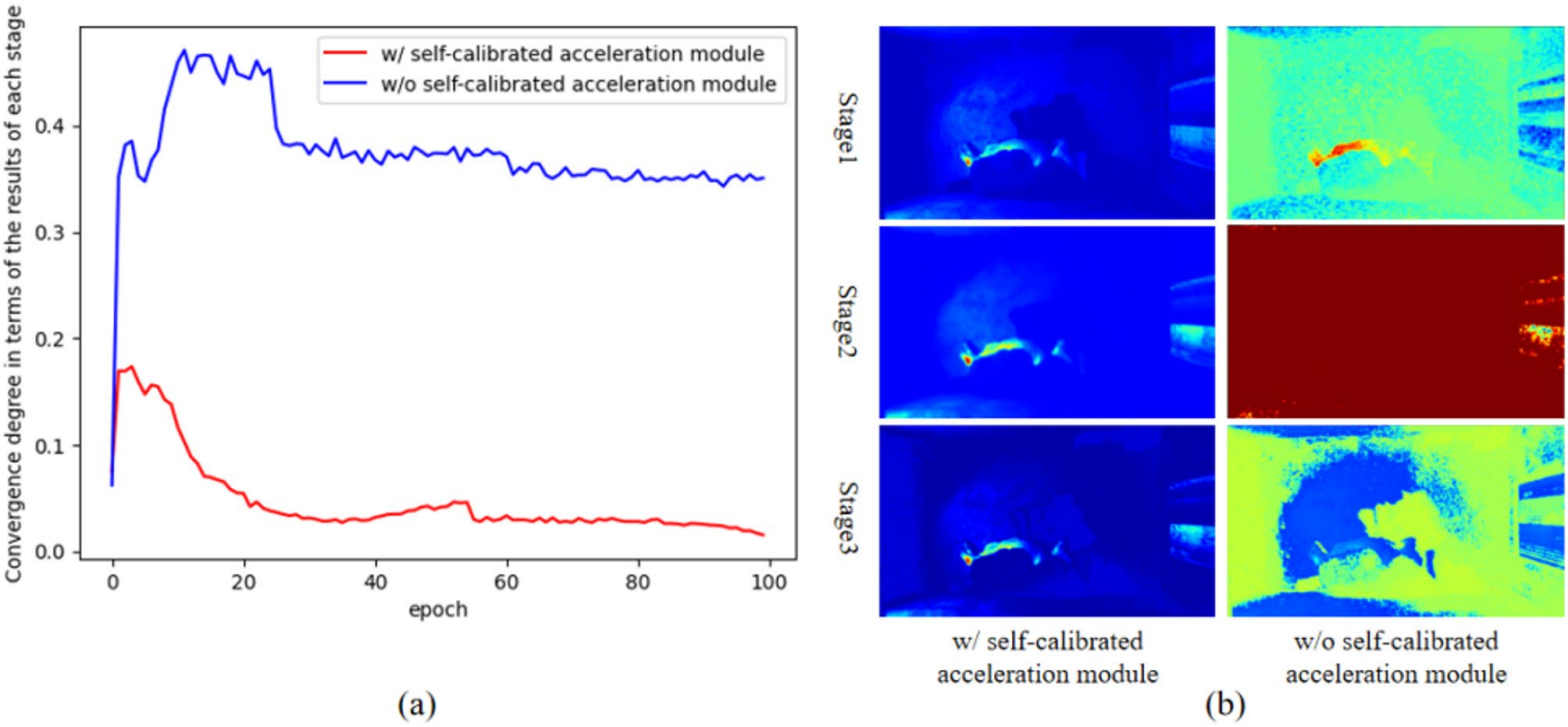
Comparing convergence degree with and without the self-calibrated module. (**a**) The pixel-wise loss among the results of each stage (**b**) the output of each stage.

Self-calibrated acceleration module aims to improve the inference speed by making the output of each stage convergent to the same one state. To verify the convergence phenomenon, 50 images were randomly selected from test images, and inputted into our model with self-calibrated acceleration module and without self-calibrated acceleration module, respectively. The pixel-wise loss among the results of each stage were calculated at each epoch. The smaller the loss, the higher convergence degree on the output of each stage. Finally, the loss among results of each stage was calculated and plotted as shown in Fig. 5(a). It can easily be seen that the self-calibrated module can effectively accelerate the convergence in each stage, which obviously surpass the backbone without self-calibrated acceleration module in efficiency. Also, we visualized the results of each stage as shown in Fig. 5(b). It is obvious that the output of each stage indeed converged to the similar state in the case of using self-calibrated acceleration module, which provides evidence that we can use a single stage for testing. This significantly improves the inference speed as shown in Table 5. (about 0.0033s/image).

#### Detail preserving loss design

In this section, we conducted the ablation study on the proposed perceptual loss $\:{L}_{sem}$and gradient loss $\:{L}_{\nabla\:}$ to demonstrate its effectiveness. Here, we studied the influence of $\:{L}_{sem}$ and $\:{L}_{\nabla\:}$, as shown in Table 3. It can be seen that the quality of enhancement on HIGRADE-1 and HIGRADE-2 metrics is improved by containing $\:{L}_{sem}$ and $\:{L}_{\nabla\:}$. Our proposed loss function achieves the best performance with an average HIGRADE-1 score of −0.1134 and HIGRADE-2 score of 0.2044, which exceed the second-best loss function (without $\:{L}_{\nabla\:}$) by 0.1199 (−0.1134 - (−0.2333)) on HIGRADE-1 and 0.0537 (0.2044 − 0.1507) on HIGRADE-2. For BRISQUE, CEIQ and ENIQA, our method containing $\:{L}_{sem}$ and $\:{L}_{\nabla\:}$ performs slightly worse than only containing one of loss terms $\:{L}_{sem}$ and $\:{L}_{\nabla\:}$, but it still outperformed the commonly used loss with loss terms $\:{L}_{f}$ and $\:{L}_{s}$. Overall, the proposed loss design achieves the best or close to the best results, demonstrating its rationality in our application scene.

**Table 3:** Evaluation the influence of loss terms. “w/o” means without.

Loss terms	HIGRADE−1↑	HIGRADE−2↑	Brisque↓	CEIQL↑	ENIQAM↓
with $\:{L}_{f}$, with $\:{L}_{s}$, w/o $\:{L}_{sem}$, w/o $\:{L}_{\nabla\:}$	−0.4212	0.0095	34.0931	2.8186	0.1699
with $\:{L}_{f}$, with $\:{L}_{s}$, with $\:{L}_{sem}$, w/o $\:{L}_{\nabla\:}$	**−0.2333**	**0.1507**	**33.6930**	2.8361	**0.1658**
with $\:{L}_{f}$, with $\:{L}_{s}$, w/o $\:{L}_{sem}$, with $\:{L}_{\nabla\:}$	−0.2465	0.1113	**33.6411**	**2.8716**	**0.1698**
with $\:{L}_{f}$, with $\:{L}_{s}$, with $\:{L}_{sem}$, with $\:{L}_{\nabla\:}$	**−0.1134**	**0.2044**	33.7508	**2.8669**	0.1712

### Comparison with state-of-the-art algorithms

#### Comparison on the image quality

In this section, we compared the proposed method with the mainstream LIE approaches, including Zero-DCE^27^ SSIENet^26^ EnlightenGAN^28^ and RUAS^29^. For each compared approach, we used the official codes and finetuned them on our dataset. The quantitative comparisons were illustrated in Table 4. It can be seen from the table that the proposed SADP model achieves the best results in most metrics. Specifically, our method performs better than other methods in terms of HIGRADE-1(+ 0.0003), HIGRADE-2 (+ 0.027) and ENIQA(−0.0059), indicating the effectiveness of the proposed method.

**Table 4:** Comparison of the proposed method with state-of-the-art unsupervised learning-based methods on quantitative results.

Methods	HIGRADE−1↑	HIGRADE−2↑	BRISQUE↓	CEIQL↑	ENIQAM↓
Zero-DCE	−1.15556	−0.0479	34.6517	2.8232	0.1798
SSIENet	−0.1988	−0.0628	36.9957	**2.9713**	0.1939
EnlightenGAN	**−0.1137**	**0.1774**	**31.5049**	2.1121	**0.1771**
RUAS	−0.2999	−0.0549	**32.6031**	2.6212	0.2040
Ours	**−0.1134**	**0.2044**	33.7508	**2.8669**	**0.1712**

**Fig. 6:**
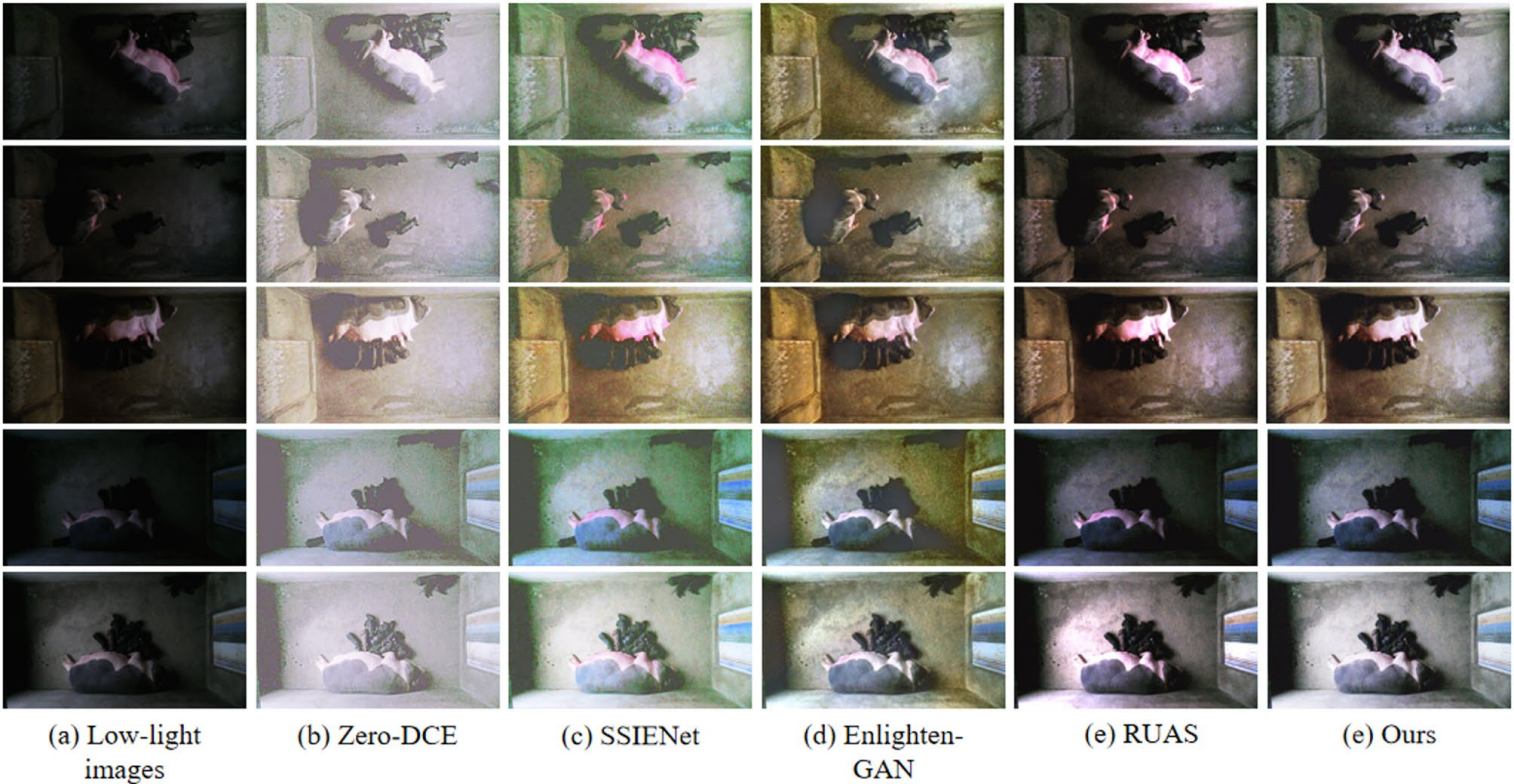
Visual comparison with the state-of-the-art unsupervised learning-based methods.

Figure 6 shows the visual comparisons with the compared approaches. It is easily to observed that some methods such as Zero-DCE and SSIENet tend to blur the details or amplify the noise, while some methods such as EnlightenGAN and RUAS tend to introduce unnatural lighting distortions and unrealistic artifacts. Obviously, the proposed SADP model produces more realistic and clear results, which can promote the manual observation and further analysis as shown in the later section.

#### Comparison on the estimated illumination

**Fig. 7:**
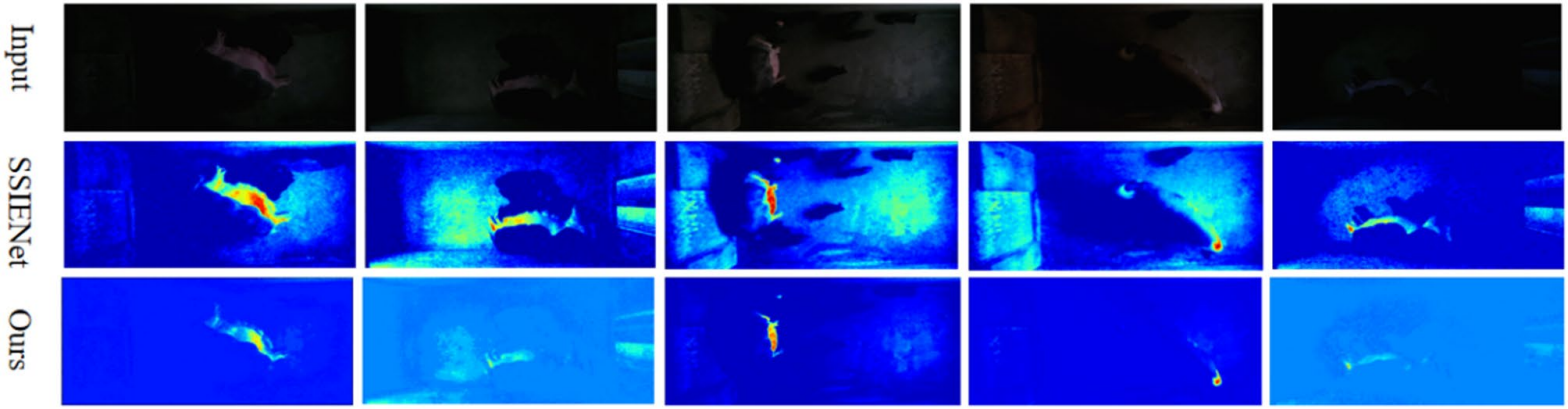
Comparing the estimated illumination with illumination learning-based network SSIENET.

Actually, the enhancement results of our SADP heavily depends on the estimated illumination because SADP was an illumination learning-based method. Here, we compared our SADP with SSIENet which was an illumination learning-based method. The estimated illumination was shown in Fig. 7. It is obvious that the estimated illumination produced by our method exhibits better smoothness property, which can help to keep our enhanced image more visually friendly.

#### Comparison on the computational efficiency

We further compared the efficiency with all the compared approaches on the test set. The model size, FLOPs, and running time of all methods were described in Table 5. All methods were tested on the same GPU device, and the comparison results confirm the superiority of our method in efficiency. The speed improved 0.013s (from 0.0163 to 0.0033) and the mode size has reduced by 0.0031 M (from 0.0034 to 0.0003) compared to RUAS.

**Table 5:** Comparison of the proposed method with state-of-the-art unsupervised learning-based methods on computational efficiency.

Methods	SIZE(M)	FLOPs(G)	TIME(S)
Zero-DCE	0.0794	41.057	0.0732
SSIENet	0.4856	2.2473	0.0323
EnlightenGAN	8.6363	130.9235	0.0403
RUAS	**0.0034**	**1.694**	**0.0163**
Ours	**0.0003**	**0.2768**	**0.0033**

### Semantic segmentation results

**Fig. 8:**
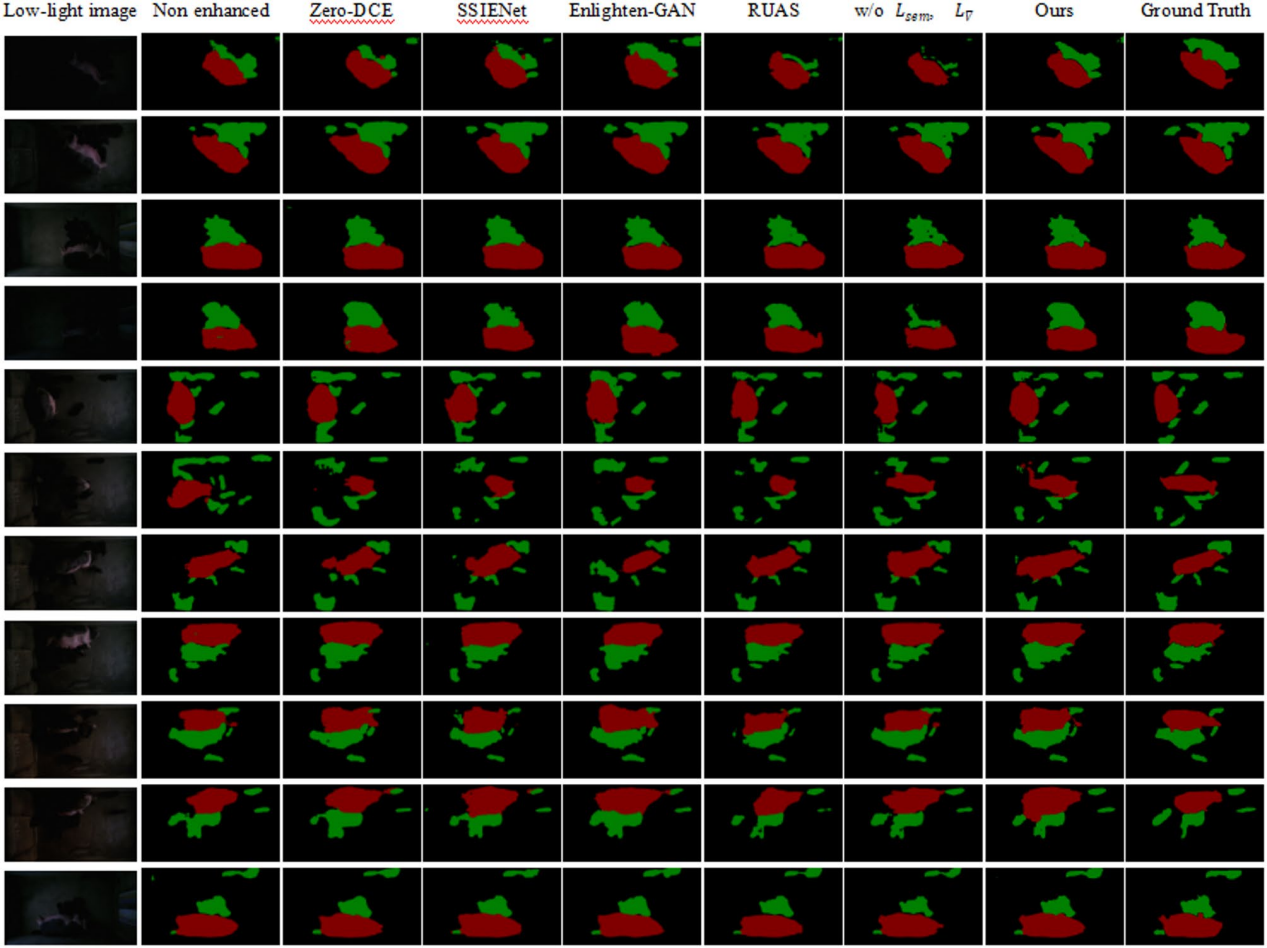
Visual results of semantic segmentation on our pig datasets.

To enhance the segmentation accurate of lactating sows and piglets under low-light conditions, we used the image enhancement as a pre-processing and then evaluated the performance improvement of our method on semantic segmentation. Specifically, we used the SEG-D1 containing 607 low-light images for training and SEG-D2 containing 300 low-light images for validation, which can be referred to Sect. 2 for the details of datasets. SADP was used as a pre-processing step to obtain clear images, then these enhanced images were sent to PSPNET. The experiment results were shown in Table 6. From Table 6, we can see that using our SADP as pre-processing improves the mean IOU from 0.8686 to 0.8872, which demonstrates that the proposed method can improve the performance of segmentation task. We also conduct segmentation experiments adopting Zero-DCE, SSIENet, EnlightenGAN and RUAS. It is obviously observed that after pre-processing by most enhancement methods, the segmentation mIOU was not be improved but decreased. This is mainly because that a lot of details were lost during enhancement processing. We argued that our method can effectively preserve the details and semantic information by adding semantic perceptual loss $\:{L}_{sem}$ terms, which can improve the performance of high-level vision tasks. From the segmentation visualization results shown in Fig. 8, it also can be seen that our method performs better in segmenting details.

**Table 6:** Quantitative results of semantic segmentation using the mainstream LIE methods on the test set.

Without pre-processing	Sow	Piglets	Background	mIoU
Segmentation on the original low-light images captured in real farrowing pens				
Segmentation results	0.9676	0.8503	0.7883	0.8686

Segmentation using image enhancement as a pre-processing step				
Method	Sow	Piglets	Background	mIoU
Zero-DCE	0.9651	0.839	0.7616	0.8551
SSIENet	0.9658	0.8332	0.7763	0.8583
EnlightenGAN	0.9652	0.8435	0.7631	0.8573
RUAS	0.9662	0.8361	0.777	0.8596
w/o L_sem_, w/o L_▿_	0.9667	0.8431	0.7797	0.8531
Ours	**0.9725**	**0.8735**	**0.8156**	**0.8872**

### Limitations and potential improvements

Although our model demonstrates promising results on testing experiments, it exhibits limitations in local illumination conditions. Our model suppresses the overexposure in uniform lighting conditions via learning residuals between the illumination and the input. However, it still likely causes localized image exposure under uneven lighting conditions. In our research conditions, especially, during nighttime, supplemental heat lamps are typically installed in farrowing houses to maintain warmth for lactating sows and piglets. These heat lamps are typically suspended above the pigs’ resting corner, providing only localized illumination. This results in severely uneven lighting conditions in the captured images. Under such conditions, our model demonstrates certain limitations.

To address the localized exposure issue, some potential improvements are introduced. For example, illumination-aware attention mechanism can be introduced into our model. First, the H component of an uneven lighting image in HSV space is obtained, and the reversed value of H component calculated by 1-H, is as the illumination-aware attention weighted to learn the model parameter. The attention weights are inversely proportional to illumination intensity, assigning lower values to well-lit regions and higher values to poorly-lit areas. In this way, the localized exposure issue may be addressed.

## Conclusion

In this paper, a self-calibrated acceleration and detail preserving low-light enhancement method (SADP) was proposed to optimize image quality and further improve the performance of semantic segmentation of lactating sows and piglets in smart livestock-farming. In SADP, a self-calibrated acceleration module was proposed to improve the computational efficiency by guiding the output of each stage to reach the same state. Later, a semantic perceptual loss term was designed to preserve details and semantic information for improving high-level visual tasks. Finally, extensive experiments including ablation studies, comparison with the state-of-the-arts low light enhancements and semantic segmentation visual task of lactating sows and piglets was conducted. Quantitative and qualitative results demonstrated the effectiveness of the proposed method on the image quality(+ 0.0003, + 0.027, −0.0059 in terms of HIGRADE-1, HIGRADE-2 and ENIQA), efficiency (−0.013s in execution time from 0.0163s to 0.0033s, −0.0031 M in mode size from 0.0034 M to 0.0003 M) and the influences to semantic segmentation task (+ 1.86% mIOU, from 86.86 to 88.72%). These results showed that the research of this work can improve visual perception and the performance of segmentation task at the scene of smart animal husbandry. This may build a good foundation for the following visual tasks and further promote the livestock breeding and production.

## Electronic supplementary material

Below is the link to the electronic supplementary material.

Supplementary Material 1

## Data Availability

Code and data for reproducing the entire contents of this article are available at https://github.com/yangaqing/SADP.

## References

[1] Guo, Q. et al. Enhanced camera-based individual pig detection and tracking for smart pig farms. *Comput. Electron. Agr*. **211**, 108009 (2023).

[2] Li, G., Shi, G. & Jiao, J. YOLOv5-KCB: A new method for individual pig detection using optimized K-Means, CA attention mechanism and a Bi-Directional feature pyramid network. *Sensors-Basel* **23**, 5242 (2023). 37299967 10.3390/s23115242 PMC10255871

[3] He, W., Mi, Y., Ding, X., Liu, G. & Li, T. Two-stream cross-attention vision transformer based on RGB-D images for pig weight Estimation. *Comput. Electron. Agr*. **212**, 107986 (2023).

[4] Yang, G. et al. Extracting cow point clouds from multi-view RGB images with an improved YOLACT + + instance segmentation. *Expert Syst. Appl.* **230**, 120730 (2023).

[5] Wang, Y. et al. E3D: an efficient 3D CNN for the recognition of dairy cow’s basic motion behavior. *Comput. Electron. Agr*. **205**, 107607 (2023).

[6] Gao, Y. et al. Recognition of aggressive behavior of group-housed pigs based on CNN-GRU hybrid model with spatio-temporal attention mechanism. *Comput. Electron. Agr*. **205**, 107606 (2023).

[7] Yun, S., Kim, J. H. & Kim, S. presented at the IEEE International Conference on Consumer Electronics (ICCE), 2011 (unpublished). (2011).

[8] Sheet, D., Garud, H., Suveer, A., Mahadevappa, M. & Chatterjee, J. Brightness preserving dynamic fuzzy histogram equalization. *IEEE T Consum. Electr.* **56**, 2475–2480 (2010).

[9] Cheng, H. & Shi, X. J. A simple and effective histogram equalization approach to image enhancement. *Digit. Signal. Process.* **14**, 158–170 (2004).

[10] Huang, S., Cheng, F. & Chiu, Y. Efficient contrast enhancement using adaptive gamma correction with weighting distribution. *IEEE T Image Process.* **22**, 1032–1041 (2012). 10.1109/TIP.2012.2226047 23144035

[11] Singh, H., Kumar, A., Balyan, L. K. & Singh, G. K. presented at the 2017 22nd International Conference on Digital Signal Processing (DSP), 2017 (unpublished).

[12] Wang, W., Sun, N. & Ng, M. K. A variational gamma correction model for image contrast enhancement. *Inverse Probl. Imag.* **13**, 461–478 (2019).

[13] Rahman, Z., Jobson, D. J. & Woodell, G. A. Retinex processing for automatic image enhancement. *J. Electron. Imaging*. **13**, 100–110 (2004).

[14] Gu, Z., Li, F., Fang, F. & Zhang, G. A novel retinex-based fractional-order variational model for images with severely low light. *IEEE T Image Process.* **29**, 3239–3253 (2019). 10.1109/TIP.2019.2958144 31841409

[15] Ren, X., Yang, W., Cheng, W. & Liu, J. LR3M: robust low-light enhancement via low-rank regularized retinex model. *IEEE T Image Process.* **29**, 5862–5876 (2020). 10.1109/TIP.2020.2984098 32286975

[16] Hao, S., Han, X., Guo, Y., Xu, X. & Wang, M. Low-light image enhancement with semi-decoupled decomposition. *IEEE T Multimedia*. **22**, 3025–3038 (2020).

[17] Nie, L. et al. Deep learning strategies with CReToNeXt-YOLOv5 for advanced pig face emotion detection. *Sci. Rep. -UK*. **14**, 1679 (2024). 10.1038/s41598-024-51755-8 PMC10799033 38242984

[18] Li, R. et al. Multi-behavior detection of group-housed pigs based on YOLOX and SCTS-SlowFast. *Comput. Electron. Agr*. **225**, 109286 (2024).

[19] Chouhan, S. S., Singh, U. P., Sharma, U. & Jain, S. Classification of different plant species using deep learning and machine learning algorithms. *Wirel. Pers. Commun.* **136**, 2275–2298 (2024).

[20] Djibrine, O. H., Ahmat, D. & Boukar, M. M. presented at the 2024 International Conference on Artificial Intelligence, Computer, Data Sciences and Applications (ACDSA), (unpublished). (2024).

[21] Ferreira, A. C. et al. Deep learning-based methods for individual recognition in small birds. *Methods Ecol. Evol.* **11**, 1072–1085 (2020).

[22] Liu, R., Ma, L., Zhang, Y., Fan, X. & Luo, Z. Underexposed image correction via hybrid priors navigated deep propagation. *IEEE T Neur Net Lear*. **33**, 3425–3436 (2021). 10.1109/TNNLS.2021.3052903 33513118

[23] Xu, K., Yang, X., Yin, B. & Lau, R. W. presented at the Proceedings of the IEEE/CVF conference on computer vision and pattern recognition, (unpublished). (2020).

[24] Zhang, Y., Zhang, J. & Guo, X. presented at the Proceedings of the 27th ACM international conference on multimedia, 2019 (unpublished).

[25] Chao, K. et al. CUI-Net: a correcting uneven illumination net for low-light image enhancement. *Sci. Rep. -UK*. **13**, 12894 (2023). 10.1038/s41598-023-39524-5 PMC10412593 37558723

[26] Zhang, Y., Di, X., Zhang, B. & Wang, C. Self-supervised Image Enhancement Network: Training with Low Light Images Only. (2020).

[27] Guo, C. et al. Zero-Reference Deep Curve Estimation for Low-Light Image Enhancement. *2020 IEEE/CVF Conference on Computer Vision and Pattern Recognition (CVPR)* (2020).

[28] Jiang, Y., Gong, X., Liu, D., Cheng, Y. & Wang, Z. EnlightenGAN: deep light enhancement without paired supervision. *IEEE T Image Process*. 10.1109/TIP.2021.3051462 (2021). 10.1109/TIP.2021.3051462 33481709

[29] Liu, R., Ma, L., Zhang, J., Fan, X. & Luo Z. presented at the Computer Vision and Pattern Recognition, (unpublished). (2021).

[30] Land, E. H. & McCann, J. J. Lightness and retinex theory. *J. Opt. Soc. Am.* **61**, 1–11 (1971). 5541571 10.1364/josa.61.000001

[31] Guo, X., Li, Y. & Ling, H. L. I. M. E. Low-light image enhancement via illumination map Estimation. *IEEE T Image Process.* **26**, 982–993 (2016). 10.1109/TIP.2016.2639450 28113318

[32] Ma, L., Ma, T., Liu, R., Fan, X. & Luo, Z. Toward Fast, Flexible, and Robust Low-Light Image Enhancement. (2022).

[33] Zhang, Y., Guo, X., Ma, J., Liu, W. & Zhang, J. Beyond brightening low-light images. *Int. J. Comput. Vis.* **129**, 1013–1037 (2021).

[34] Simonyan, K. & Zisserman, A. Very deep convolutional networks for Large-Scale image recognition. *Comp. Sci.*10.48550/arXiv.1409.1556 (2014).

[35] Zhao, H., Shi, J., Qi, X., Wang, X. & Jia, J. Pyramid scene parsing network. *IEEE Comput. Society*. 10.1109/CVPR.2017.660 (2016).

[36] Kundu, D., Ghadiyaram, D., Bovik, A. C. & Evans, B. L. No-reference quality assessment of tone-mapped HDR pictures. *IEEE T Image Process.* **26**, 2957–2971 (2017). 10.1109/TIP.2017.2685941 28333633

[37] Mittal, P., Saini, R., Varghese, J. & Jain, N. A technical review of no-reference image quality assessment algorithms for contrast distorted images. *J. Eng. Res.* **11**. 10.36909/jer.11885 (2023).

[38] Paszke, A. et al. Automatic differentiation in pytorch. (2017).

