# Supplementary material for: Self-calibrated acceleration and detail preserving for semantic segmentation of lactating sows and piglets under low-light conditions
Source: Sci Rep. 2025 Jul 3;15:23795. doi: 10.1038/s41598-025-09146-0 (PMC12229340; doi:10.1038/s41598-025-09146-0)
Supplement: Supplementary file 1 — Supplementary Material 1 [file 41598_2025_9146_MOESM1_ESM.docx]

**Ethics declarations**

Since the research work of this paper only covered the observational field studies, which did not involve any type of interaction with animals or manipulation of the environment. Therefore, this study did not require ethical approval according to rules of Institutional Animal Care and Use Committee. Confirmation that these pigs were not handled by the authors during the study. Confirmation that all experimental protocols have been approved by Lejiazhuang farm in Foshan City, Guangdong Province, China, where the video data were collected. Confirm that all methods were performed in accordance with relevant guidelines and regulations.
